# Development and validation of machine-learning models for the difficulty of retroperitoneal laparoscopic adrenalectomy based on radiomics

**DOI:** 10.3389/fendo.2023.1265790

**Published:** 2023-11-16

**Authors:** Shiwei Sun, Wei Yao, Yue Wang, Peng Yue, Fuyu Guo, Xiaoqian Deng, Yangang Zhang

**Affiliations:** ^1^Third Hospital of Shanxi Medical University, Shanxi Bethune Hospital, Shanxi Academy of Medical Sciences, Tongji Shanxi Hospital, Taiyuan, China; ^2^Shanxi Bethune Hospital, Shanxi Academy of Medical Sciences, Tongji Shanxi Hospital, Third Hospital of Shanxi Medical University, Taiyuan, China; ^3^Tongji Hospital, Tongji Medical College, Huazhong University of Science and Technology, Wuhan, China

**Keywords:** adrenal tumor, laparoscopy, retroperitoneal space, machine learning, radiomics, random forest, extreme gradient boosting

## Abstract

**Objective:**

The aim is to construct machine learning (ML) prediction models for the difficulty of retroperitoneal laparoscopic adrenalectomy (RPLA) based on clinical and radiomic characteristics and to validate the models.

**Methods:**

Patients who had undergone RPLA at Shanxi Bethune Hospital between August 2014 and December 2020 were retrospectively gathered. They were then randomly split into a training set and a validation set, maintaining a ratio of 7:3. The model was constructed using the training set and validated using the validation set. Furthermore, a total of 117 patients were gathered between January and December 2021 to form a prospective set for validation. Radiomic features were extracted by drawing the region of interest using the 3D slicer image computing platform and Python. Key features were selected through LASSO, and the radiomics score (Rad-score) was calculated. Various ML models were constructed by combining Rad-score with clinical characteristics. The optimal models were selected based on precision, recall, the area under the curve, F1 score, calibration curve, receiver operating characteristic curve, and decision curve analysis in the training, validation, and prospective sets. Shapley Additive exPlanations (SHAP) was used to demonstrate the impact of each variable in the respective models.

**Results:**

After comparing the performance of 7 ML models in the training, validation, and prospective sets, it was found that the RF model had a more stable predictive performance, while xGBoost can significantly benefit patients. According to SHAP, the variable importance of the two models is similar, and both can reflect that the Rad-score has the most significant impact. At the same time, clinical characteristics such as hemoglobin, age, body mass index, gender, and diabetes mellitus also influenced the difficulty.

**Conclusion:**

This study constructed ML models for predicting the difficulty of RPLA by combining clinical and radiomic characteristics. The models can help surgeons evaluate surgical difficulty, reduce risks, and improve patient benefits.

## 1 Introduction

Adrenal tumors (ATs) are a rare type of tumor that usually occurs in the cortex or medulla of the adrenal gland (1). Depending on their type and size, these tumors can be benign or malignant (2). ATs can cause many symptoms, including high blood pressure, palpitations, headaches, insomnia, anxiety, and obesity (3). In some cases, these symptoms may be mistaken for symptoms of other diseases, so further testing is needed to determine the diagnosis (4–6).

Treatment for AT includes surgery, radiation therapy, and chemotherapy. Surgery is the most common treatment method and can altogether remove the tumor (7). The gold standard treatment for AT is laparoscopic surgery, which can be divided into two main approaches: transperitoneal laparoscopic adrenalectomy (TPLA) and retroperitoneal laparoscopic adrenalectomy (RPLA) (8). The RPLA involves entering the retroperitoneal cavity through laparoscopic surgery, avoiding interference with abdominal organs, and reducing surgical trauma and recovery time. Compared with traditional open surgery, this technique has fewer complications and faster recovery (6, 9, 10).

In the field of medicine, machine learning (ML) has wide-ranging applications (3). For example, ML can be used for medical image recognition to help doctors diagnose diseases. It can also be used to predict the health status of patients, assisting doctors to develop better treatment plans. In addition, ML can be used for drug development and clinical trials to speed up the development and launch of new drugs (11). For example, a study has used ML to differentiate between adrenal pheochromocytoma and adrenocortical adenoma (12).

Radiomics is an emerging field of medicine that combines computer science, mathematics, and medical imaging to understand better and diagnose diseases (13, 14). Radiomics analyzes large amounts of medical imaging data to extract useful information, helping doctors make more accurate diagnoses and treatment decisions (15).

This study aimed to collect data retrospectively from patients with AT who underwent RPLA at Shanxi Bethune Hospital from August 2014 to December 2020. The study utilized ML to analyze their clinical and radiomics features and develop a predictive model for the difficulty of RPLA. The goal was to improve preoperative preparation, reduce surgical risks, and enhance patient benefits.

## 2 Method

### 2.1 General information

**Figure 1:**
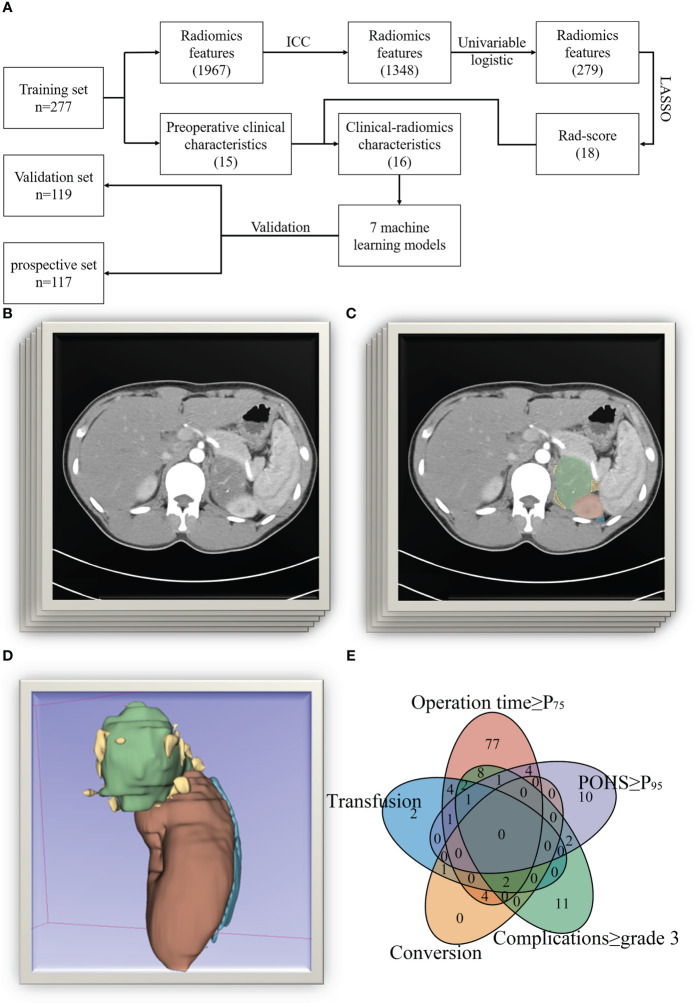
The process of this study. (**A** Flowchart of this study; **(B)** Original CT images; **(C)** Drawing of regions of interest [ROIs]; **(D)** 3D reconstruction of the ROIs; **(E)** Venn plot of the reasons for the difficulty of surgery).

We retrospectively collected data from patients with AT treated at Shanxi Bethune Hospital between August 2014 and December 2020. A model was established using this data and prospectively validated with AT patients treated from January 2021 to December 2021. Inclusion criteria: 1) abdominal Computed Tomography (CT) examination confirming the presence of an AT within 15 days before surgery, 2) preoperative routine laboratory tests to determine the hormonal activity of AT, and 3) treatment of AT with laparoscopic surgery. Exclusion criteria: 1) patients who did not undergo surgery, 2) patients who underwent multiple surgeries concurrently, 3) patients treated for AT with other surgical methods, and 4) patients with incomplete preoperative radiological examination. A total of 396 patients were included in the study, and an additional 117 patients were collected for prospective validation (**Figure 1A**). All surgical procedures are performed by a cohesive team within the same department at a single center, led by an expert surgeon with 35 years of experience.

### 2.2 Research method

Referring to previous studies (9, 10, 16–20) and combining practical experience, we defined cases with serious surgical difficulty if any of the following conditions were met: 1) operation time ≥ P_75_ (150 min), 2) intraoperative injury to organs or vessels requiring blood transfusion, 3) conversion from minimally invasive to open surgery, 4) postoperative complications of Clavien-Dindo classification (21) greater than or equal to grade 3, 5) Postoperative hospital stay ≥ P_95_ (15 days).

**Figure 2:**
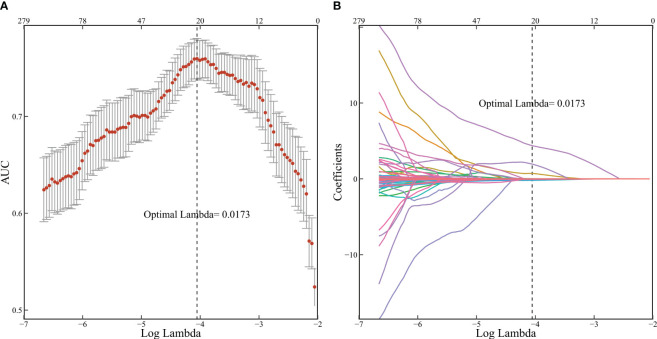
Radiomic features selected by LASSO. (**A** 10-fold cross-validation corresponding AUC results; **(B)** 279 feature screening adjoint coefficient changes).

The Siemens Somatom Definition Flash or Force dual-source CT scanner (manufactured by Siemens AG in Munich, Germany) was utilized for the purpose of scanning and reconstructing thin-slice images. DICOM format was used to export the images. Using the 3D Slicer image computing platform (version 5. 0. 2), two radiology-trained urologists independently identified the region of interest (ROI) in arterial-enhanced images. Python 3. 7. 1 (Python Software Foundation) was utilized to extract radiomics features from the images (**Figures 1B–D**). The included image types are Original, Wavelet, Laplacian of Gaussian, Square, Square Root, Logarithm, Exponential, Gradient, Local Binary Pattern 2D, and Local Binary Pattern 3D. The feature types consisted of First Order Features, Shape Features (3D), Shape Features (2D), Gray Level Co-occurrence Matrix (GLCM) Features, Gray Level Size Zone Matrix (GLSZM) Features, Gray Level Run Length Matrix (GLRLM) Features, Neighboring Gray Tone Difference Matrix (NGTDM) Features, Gray Level Dependence Matrix (GLDM) Feature. A total of 1967 radiomics features were extracted (**Figure 2**). Data on patients’ clinical conditions and treatment were obtained from the computerized physician order entry and medical record management system (Winning Health Technology Group Co., Ltd., Shanghai, China).

Patients were randomly divided into a training set and a validation set at a ratio of 7:3. The training set was used for model construction, and the validation set was used for model validation.

### 2.3 Statistical methods

Data were further analyzed using R 4. 2. 3 (Vienna Statistical Computing Foundation, Austria). All continuous variables were non-normally distributed and were presented as median [interquartile range]; categorical variables were presented as frequency and percentage (%). Analysis of variance (ANOVA) was used to compare differences between sets in the training set, validation set, and prospective set. The consistency of the regions of interest (ROIs) drawn by the two urologists was evaluated using the intraclass correlation coefficient (ICC), excluding features with a correlation below 0. 75. Radiomics features were subjected to univariable logistic regression analysis using the “glmnet” package. Factors with a P-value greater than 0. 05 were considered unrelated and subsequently excluded. Key features were selected using the Least Absolute Shrinkage and Selection Operator (LASSO), and the radiomics score (Rad-score) was calculated based on the results. Using the “mlr3” package, seven ML models were developed by combining the Rad-score with clinical characteristics. These models included Classification and Regression Trees (CART), K-Nearest Neighbors (KNN), LASSO, Naive Bayes (NB), Random Forest (RF), Support Vector Machine (SVM), and Extreme Gradient Boosting (xGBoost). The optimal models were selected based on precision, recall, area under the curve (AUC), F1 score, calibration curve, Receiver Operating Characteristic (ROC) curve, and decision curve analysis (DCA) in the training set, validation set, and prospective set. Shapley Additive exPlanations (SHAP) value demonstrated the impact of each variable in the respective model (22).

## 3 Results

### 3.1 General information

A total of 396 patients were included in the study. Patients were randomly divided into a training set and a validation set at a ratio of 7:3. Baseline patient characteristics are shown in **Table 1**. A total of 130 patients were considered to have high surgical difficulty due to meeting one or more criteria, with specific reasons shown in **Figure 1E**. An additional 117 patients were collected and regarded as a prospective set. ANOVA showed no statistically significant differences in baseline characteristics between sets.

**Table 1:** Baseline clinical and radiomics characteristics of patients.

Characteristics	Training set	Validation set	Prospective set	F	P
Radiomics characteristics					
Rad score	8.87[8.76,9.00]	8.87[8.74,8.97]	8.87[8.77,8.97]	1.030	0.358
Clinical characteristics					
Preoperative					
Gender				0.732	0.482
Male	125(45.1)	53(44.5)	60(51.3)		
Female	152(54.9)	66(55.5)	57(48.7)		
Age (year)	50.00[40.00,59.00]	51.00[42.00,58.00]	51.00[39.00,58.00]	0.047	0.954
BMI (kg/m^2^)	24.72[22.68,27.51]	24.57[23.10,27.36]	25.95[23.44,28.32]	2.430	0.089
Side				1.130	0.324
Left	161(58.1)	77(64.7)	65(55.6)		
Right	116(41.9)	42(35.3)	52(44.4)		
Hypertension				0.417	0.660
No	58(20.9)	27(22.7)	21(17.9)		
Yes	219(79.1)	92(77.3)	96(82.1)		
Diabetes mellitus				1.446	0.236
No	229(82.7)	92(77.3)	89(76.1)		
Yes	48(17.3)	27(22.7)	28(23.9)		
Scoliosis				1.108	0.331
No	264(95.3)	113(95.0)	115(98.3)		
Yes	13(4.7)	6(5.0)	2(1.7)		
Coronary disease				1.673	0.189
No	249(89.9)	107(89.9)	98(83.8)		
Yes	28(10.1)	12(10.1)	19(16.2)		
Cerebral infarction				0.965	0.382
No	254(91.7)	104(87.4)	107(91.5)		
Yes	23(8.3)	15(12.6)	10(8.5)		
Hyperlipidemia				0.393	0.675
No	245(88.4)	108(90.8)	102(87.2)		
Yes	32(11.6)	11(9.2)	15(12.8)		
History of malignancy				2.129	0.120
No	270(97.5)	113(95.0)	109(93.2)		
Yes	7(2.5)	6(5.0)	8(6.8)		
History of operation				0.436	0.647
No	202(72.9)	84(70.6)	80(68.4)		
Yes	75(27.1)	35(29.4)	37(31.6)		
Infectious disease				0.262	0.769
No	272(98.2)	118(99.2)	115(98.3)		
Yes	5(1.8)	1(0.8)	2(1.7)		
Previous antiplatelet use				2.567	0.078
No	268(96.8)	116(97.5)	108(92.3)		
Yes	9(3.2)	3(2.5)	9(7.7)		
Hb (g/L)	137.0[126.0,145.0]	138.0[127.5,147.0]	137.0[127.0,146.0]	0.065	0.937
Intraoperative and postoperative					
Range				0.475	0.622
Partial	253(91.3)	105(88.2)	105(89.7)		
Radical	24(8.7)	14(11.8)	12(10.3)		
Operation time (min)	110.0[90.0,150.0]	110.0[90.0,149.0]	98.0[76.0,130.0]	4.130	0.017
Blood loss (ml)	20.0[0.0,50.0]	20.0[0.0,50.0]	15.0[0.0,25.0]	0.264	0.768
POHS	8.0[6.0,9.0]	7.0[6.5,9.0]	6.0[2.0,7.0]	15.966	<0.001
Conversion				0.558	0.573
No	273(98.6)	116(97.5)	116(99.1)		
Yes	4(1.4)	3(2.5)	1(0.9)		
Transfusion				0.078	0.925
No	268(96.8)	115(96.6)	114(97.4)		
Yes	9(3.2)	4(3.4)	3(2.6)		
Complications				0.122	0.885
None	248(89.5)	107(89.9)	105(89.7)		
1	9(3.2)	2(1.7)	4(3.4)		
2	2(0.7)	1(0.8)	0(0.0)		
3a	1(0.4)	0(0.0)	3(2.6)		
3b	0(0.0)	1(0.8)	0(0.0)		
4	17(6.1)	8(6.7)	5(4.3)		
Pathology				1.330	0.265
NFAT	107(38.6)	51(42.9)	38(32.5)		
Aldosteronoma	79(28.5)	33(27.7)	43(36.8)		
Cushing’s syndrome	25(9.0)	16(13.4)	17(14.5)		
Paraganglioma	26(9.4)	7(5.9)	7(6.0)		
Myelolipoma	10(3.6)	5(4.2)	4(3.4)		
Cyst	13(4.7)	3(2.5)	3(2.6)		
Malignant tumor	5(1.8)	1(0.8)	2(1.7)		
Ganglioneuroma	2(0.7)	2(1.7)	2(1.7)		
Others	10(3.6)	1(0.8)	1(0.9)		

BMI, body mass index; NFAT, non-function adrenal tumor; Others(pathology) include eosinophil tumor, teratoma, schwannoma, hematoma, tuberculoma, foreign body granuloma, retroperitoneal bronchial cyst, hemangioma.

### 3.2 Radiomics feature selection

Consistency was assessed using ICC, excluding 619 radiomics features with consistency lower than 0.75 to eliminate the interference of human factors on the model. Univariable logistic analysis was performed, excluding 1069 variables with P > 0.05. The remaining 279 features underwent dimensionality reduction using LASSO and ten-fold cross-validation. As the logarithm of the harmonic parameter (λ) changed on the horizontal axis, the AUC on the vertical axis also changed. The corresponding number of selected variables is shown in **Figure 2A**. A risk factor classifier was constructed using LASSO (**Figure 2B**), with 18 features selected (**Table 2**). The optimal λ value was 0.0173, with a logarithm of -4.055.

**Table 2:** Comparison of machine learning model performance.

Model	Set	AUC	Sensitivity	Specificity	Precision	F1-score
CART	Training	0.866	0.667	0.901	0.780	0.719
CART	Validation	0.421	0.235	0.776	0.296	0.262
CART	Prospective	0.356	0.393	0.809	0.393	0.393
KNN	Training	0.983	0.698	0.978	0.944	0.802
KNN	Validation	0.579	0.147	0.835	0.263	0.189
KNN	Prospective	0.588	0.286	0.798	0.308	0.296
LASSO	Training	0.824	0.417	0.956	0.833	0.556
LASSO	Validation	0.631	0.294	0.918	0.588	0.392
LASSO	Prospective	0.720	0.321	0.921	0.562	0.409
NB	Training	0.779	0.458	0.834	0.595	0.518
NB	Validation	0.696	0.441	0.776	0.441	0.441
NB	Prospective	0.662	0.536	0.730	0.385	0.448
RF	Training	0.994	0.885	0.994	0.988	0.934
RF	Validation	0.681	0.353	0.835	0.462	0.400
RF	Prospective	0.724	0.536	0.820	0.484	0.508
SVM	Training	0.917	0.688	0.950	0.880	0.772
SVM	Validation	0.677	0.294	0.847	0.435	0.351
SVM	Prospective	0.617	0.286	0.775	0.286	0.286
xGBoost	Training	0.914	0.750	0.917	0.828	0.787
xGBoost	Validation	0.615	0.353	0.706	0.324	0.338
xGBoost	Prospective	0.710	0.500	0.764	0.400	0.444
Clinical	Training	0.660	0.719	0.514	0.439	0.545
Clinical	Validation	0.621	0.735	0.431	0.357	0.481
Clinical	Prospective	0.658	0.821	0.461	0.324	0.465

Based on the LASSO results (**Table 3**), Rad-score was calculated. The specific calculation formula is provided in the **Supplementary Data****.**

**Table 3:** Radiomic features selected by LASSO.

Image types	Feather types	Feathers	Coefficients
Logarithm	GLCM	Imc1	-0.197
Square	GLSZM	SizeZoneNonUniformityNormalized	-0.134
Wavelet.LLH	Firstorder	Mean	-0.060
Wavelet.LLL	Firstorder	Kurtosis	-0.049
Original	GLSZM	SizeZoneNonUniformityNormalized	-0.034
Log.sigma.4.mm.3D	Firstorder	90Percentile	-0.012
Log.sigma.5.mm.3D	Firstorder	90Percentile	-0.001
Squareroot	GLSZM	LargeAreaEmphasis	9.721*10^-11^
Wavelet.LLL	GLDM	DependenceEntropy	0.001
Original	Shape	Maximum3DDiameter	0.003
Wavelet.HHL	GLRLM	LongRunLowGrayLevelEmphasis	0.006
Wavelet.HLL	GLRLM	LongRunLowGrayLevelEmphasis	0.011
Logarithm	Firstorder	InterquartileRange	0.014
Log.sigma.5.mm.3D	Firstorder	Kurtosis	0.022
Log.sigma.4.mm.3D	GLDM	LowGrayLevelEmphasis	0.031
Lbp.3d.k	GLDM	DependenceNonUniformityNormalized	0.714
Wavelet.HLH	GLCM	DifferenceEntropy	1.949
Wavelet.HLL	GLCM	Imc1	4.384

### 3.3 Construction of clinical-radiomics machine learning models

**Figure 3:**
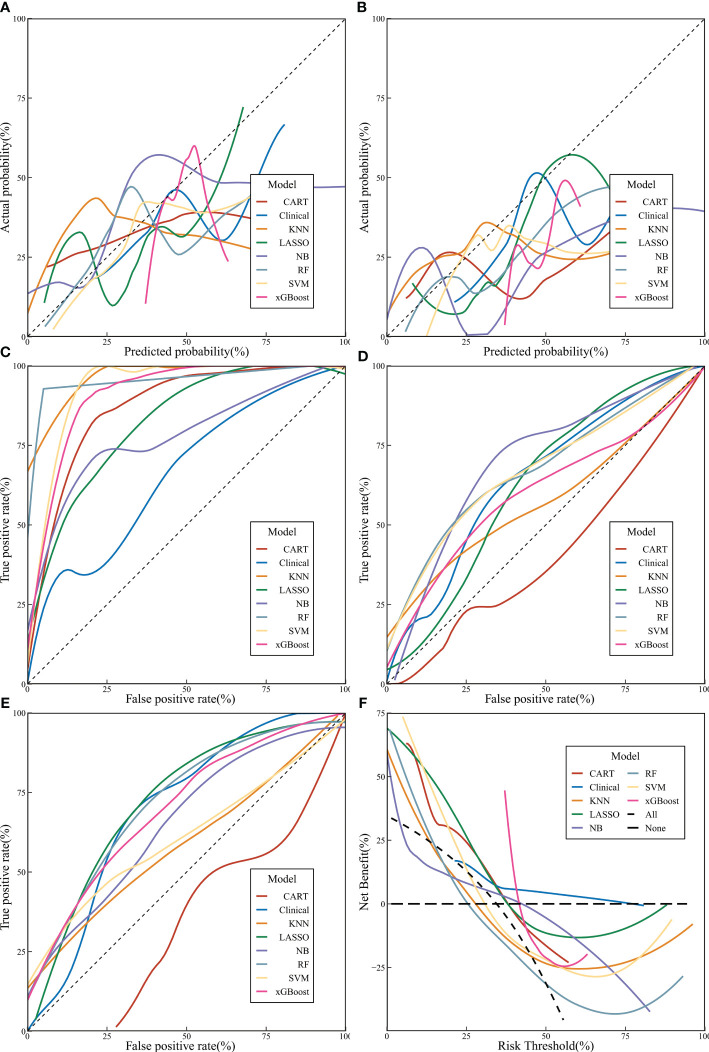
The performance of machine learning models. (**A** Calibration curve of validation set; **(B)** Calibration curve of prospective set; **(C)** Receiver operating characteristic [ROC] curves of machine learning models in training set; **(D)** ROC curves of machine learning models in validation set; **(E)** ROC curves of machine learning models in prospective set; **(F)** decision curve analysis curves of machine learning models).

The Rad-score calculated above was combined with clinical characteristics to construct CART, KNN, LASSO, NB, RF, SVM, and xGBoost models (**Table 2**). Additionally, we constructed a clinical model for comparison using stepwise logistic regression based on the clinical characteristics of the patients. All models demonstrated high predictive ability in the training set, with acceptable consistency in the validation and prospective sets (**Figures 3A, B**).

A comprehensive evaluation of precision, F1 score, ROC curve, and AUC values in the training, validation, and prospective sets revealed that the RF model had a more stable predictive performance, followed by xGBoost and LASSO (**Figures 3C–E**). According to DCA, it is evident that xGBoost can significantly benefit patients (**Figure 3F**).

**Figure 4:**
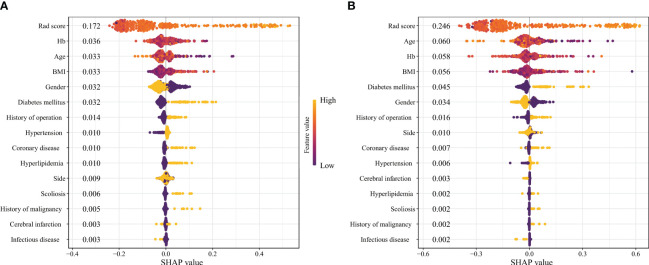
The SHAP plots of machine learning models. (**A** Random Forest; **(B)** Extreme Gradient Boosting).

The SHAP value and SHAP plot were used to display the importance of each variable in the RF and xGBoost models. According to the SHAP, the variable importance of the two models is similar, and both can reflect that the Rad-score has the most significant impact. At the same time, other clinical characteristics such as Hemoglobin (Hb), age, Body Mass Index (BMI), gender, and diabetes mellitus also influenced the difficulty (**Figure 4**).

## 4 Discussion

AT has become a hot topic in the medical field, and surgery is the primary treatment method. TPLA and RPLA were proposed in 1992 (8, 23), respectively, and have continuously improved. The advantage of RPLA is that it results in less surgical trauma and bleeding, faster recovery, and fewer complications. Moreover, it is also suitable for some cases that traditional surgery finds challenging, such as obesity and complex AT. There are some relatively objective analysis systems for the surgical difficulty of TPLA, while there is less analysis on RPLA (10, 17).

This study retrospectively analyzed 396 patients who underwent RPLA for AT. The LASSO analysis of radiomics features was used to calculate the Rad-score. By combining the Rad-score with preoperative clinical characteristics, ML models such as CART, KNN, LASSO, NB, RF, SVM, and xGBoost were constructed and compared. It was found that RF had a more stable prediction accuracy, while xGBoost could bring more significant benefits to patients. The ML model suggested that in addition to the most influential Rad-score, the clinical characteristics such as Hb, age, BMI, gender, and diabetes mellitus also greatly influenced surgical difficulty. Through the validation of the validation set and prospective set, it was found that the ML models had high predictive ability. Through the comprehensive comparison of different models, it was found that the RF model exhibits the best prediction performance, thus making it our recommended model. Furthermore, in comparison to clinical models in previous study (16), our RF model exhibited superiority as evidenced by 2000 Bootstrap tests (D = 7.155, P < 0.001). The discrimination power of models can be effectively compared using two measures: the Net Classification Index (NRI) and the Integrated Discrimination Improvement (IDI). In comparison to previous studies, the RF model in this study demonstrated an NRI of 0.308 (95% CI: 0.194-0.422, p < 0.001) and an IDI of 0.165 (95% CI: 0.119-0.210, p < 0.001).

The Rad-score calculated based on LASSO significantly impacts the surgical difficulty of RPLA. When performing univariate logistic regression, 279 features were statistically significant. After LASSO, 18 variables were retained and used to construct the Rad-score. The final retained variables included “Shape Features” like “Maximum3DDiameter”. Moreover, many studies have generally confirmed that the maximum diameter of the tumor is an essential factor affecting the difficulty of removing AT (9, 10, 16–20). In addition, “First Order Features”, which are linearly correlated with the CT value of the tumor, such as “90Percentile” were also included. Malignant and benign AT have different degrees of enhancement during arterial enhancement, which increases the risk of bleeding during surgery (18, 24, 25). It may also be because lipid-rich AT has lower CT values and requires more attention during surgery to prevent breaking the capsule, which prolongs the operation time (9, 16, 20). “DifferenceEntropy” in “GLCM Features” measures the randomness or complexity of differences between pixel intensity values. It was included because malignant tumors, such as metastases, exhibit more randomness or complexity between pixel intensity values, while their removal is more challenging than benign tumors (18).

Some clinical characteristics of patients also affect the difficulty of RPLA. Patients with diabetes mellitus are more likely to have perirenal fat adhesions, which affect surgical difficulty (26). Studies by Chen (17) and Takeda (27) have also shown that diabetes mellitus significantly affects it. Some studies also suggest that a history of hypertension and coronary heart disease affects surgical difficulty (28, 29). BMI is used to assess the degree of obesity and also affects it. However, it mainly reflects the overall body fat composition, while the distribution of visceral fat, especially perirenal fat, may differ (9, 16). Therefore, there is still controversy over BMI prediction of surgical difficulty. Some studies believe that measuring visceral fat would be more accurate (10, 25, 29).

Hb reflects the patient’s blood reserve and blood oxygen reserve situation (30). If it is too low, it will affect the surgery. Age affects almost all tumor surgeries and prognoses because older patients often have poorer nutrition and tolerance. Moreover, diseases tend to be more malignant in older patients (31, 32). In addition, some researchers believe that males may have more dangerous lifestyles (such as smoking), and there are differences in hormone levels between men and women, which may lead to poorer physical conditions and more incredible surgical difficulty in male patients (33, 34).

This study established ML models for predicting the difficulty of RPLA based on preoperative radiomics and clinical characteristics. It was validated internally and prospectively to prove that the ML models can significantly improve patients’ net benefit rate.

There needs to be more accurate prediction models for the difficulty of RPLA. The innovation of this study lies in combining ML with radiomics to analyze the risk factors for the difficulty of RPLA and establish prediction models for it, then conduct internal validation and prospective validation to make the model more meaningful. Moreover, this study is currently one of the largest cohorts using radiomics to predict the difficulty of RPLA.

The prospects of this study include: external validation to confirm its stability and accuracy further; using radiomics to analyze the tumor’s surrounding environment while analyzing AT and optimizing the model through more ML algorithms. Some studies have proposed that magnetic resonance imaging has multiple weighted sequences, which may have better effects when applied to radiomics than CT. Although the accuracy of this study’s models is high, the time cost of drawing ROI is high. If further promotion or clinical transformation is needed, combining deep learning to train artificial intelligence to draw ROI is necessary. Some studies have successfully trained artificial intelligence to draw ROIs for pancreatic duct tumors and predicted lymph node metastasis and prognosis based on them. Its sensitivity and specificity are superior to clinical and radiomics models (35).

In conclusion, Rad-score, Hb, age, BMI, gender, and diabetes mellitus affect RPLA surgical difficulty. The ML prediction model established based on patient clinical characteristics and Rad-score using RF and xGBoost has good predictive performance. Through the above model, surgeons can effectively evaluate the difficulty of RPLA, thereby reducing surgical risks and improving patient benefits.

## Data availability statement

The raw data supporting the conclusions of this article will be made available by the authors, without undue reservation.

## Ethics statement

The studies involving humans were approved by Ethics Committee of the Shanxi Bethune Hospital. The studies were conducted in accordance with the local legislation and institutional requirements. Written informed consent for participation was not required from the participants or the participants’ legal guardians/next of kin in accordance with the national legislation and institutional requirements.

## Author contributions

SS: Conceptualization, Data curation, Methodology, Project administration, Validation, Visualization, Writing – original draft. WY: Data curation, Validation, Visualization, Writing – original draft. YW: Data curation, Validation, Writing – original draft. PY: Data curation, Writing – original draft. FG: Data curation, Writing – original draft. XD: Data curation, Writing – original draft. YZ: Conceptualization, Methodology, Project administration, Supervision, Writing – review & editing.
