# Supplementary material for: Development and validation of machine-learning models for the difficulty of retroperitoneal laparoscopic adrenalectomy based on radiomics
Source: Front Endocrinol (Lausanne). 2023 Nov 16;14:1265790. doi: 10.3389/fendo.2023.1265790 (PMC10687448; doi:10.3389/fendo.2023.1265790)
Supplement: Supplementary file 1 [file Table_1.docx]

Rad-score=-0.197*logarithm_glcm_Imc1-0.134*square_glszm_SizeZoneNonUniformityNormalized-0.06*wavelet.LLH_firstorder_Mean-0.049*wavelet.LLL_firstorder_Kurtosis-0.034*original_glszm_SizeZoneNonUniformityNormalized-0.012*log.sigma.4.mm.3D_firstorder_90Percentile-0.001*log.sigma.5.mm.3D_firstorder_90Percentile+9.721*10^-11^*squareroot_glszm_LargeAreaEmphasis+0.001*wavelet.LLL_gldm_DependenceEntropy+0.003*original_shape_Maximum3DDiameter+0.006*wavelet.HHL_glrlm_LongRunLowGrayLevelEmphasis+0.011*wavelet.HLL_glrlm_LongRunLowGrayLevelEmphasis+0.014*logarithm_firstorder_InterquartileRange+0.022*log.sigma.5.mm.3D_firstorder_Kurtosis+0.031*log.sigma.4.mm.3D_gldm_LowGrayLevelEmphasis+0.714*lbp.3D.k_gldm_DependenceNonUniformityNormalized+1.949*wavelet.HLH_glcm_DifferenceEntropy+4.384*wavelet.HLL_glcm_Imc1+7.260
